# Right ventricular failure after LVAD support: A challenging case of bridge to heart transplantation in end-stage dilated cardiomyopathy

**DOI:** 10.2478/jccm-2025-0038

**Published:** 2026-01-30

**Authors:** Horatiu Suciu, Emanuel-David Anitei, Paul Calburean, Marius Mihai Harpa

**Affiliations:** Department of Surgery IV, George Emil Palade University of Medicine, Pharmacy, Science, and Technology of Targu Mures,Romania; Department of Cardiovascular Surgery, Emergency Institute for Cardiovascular Diseases and Transplantation Targu Mures, Romania; Department of Cardiology, Emergency Institute for Cardiovascular Diseases and Transplantation Targu Mures, Romania

**Keywords:** mechanical circulatory support, heart transplant, bridge to transplant, right ventricle failure

## Abstract

**Introduction:**

End-stage heart failure due to dilated cardiomyopathy remains a major indication for advanced mechanical circulatory support and heart transplantation. Left ventricular assist devices have emerged as a vital bridge to transplant, improving survival and functional status. However, right ventricular failure following LVAD implantation is a significant and potentially fatal complication, requiring careful management to optimize outcomes.

**Case presentation:**

We present the case of a 46-year-old male with post-myocarditis dilated cardiomyopathy, severely reduced left ventricular ejection fraction (21%), severe functional mitral and tricuspid regurgitation, and NYHA class IV heart failure. Despite optimal medical therapy, including inotropic support, the patient progressed to multiorgan dysfunction necessitating renal replacement therapy. A HeartMate 3 LVAD was implanted as a bridge to transplantation. The postoperative course was complicated by severe right ventricular failure, requiring prolonged inotropic support and careful hemodynamic management. Despite these challenges, the patient successfully underwent orthotopic heart transplantation. His postoperative evolution was favorable, with stable graft function and good clinical recovery documented during follow-up.

**Conclusion:**

Right ventricular failure remains a major complication following LVAD implantation, significantly impacting outcomes. While LVADs have revolutionized the management of end-stage heart failure, heart transplantation continues to represent the definitive therapy offering superior long-term survival.

## Introduction

Heart failure (HF) represents a major global health burden, with an estimated 64.3 million people affected worldwide as of 2017, a number expected to increase due to improved survival after HF diagnosis and the aging population [1]. In European countries, the median incidence of HF is approximately 3.2 cases per 1000 person-years, with a median prevalence of 17.2 cases per 1000 individuals [2]. In the United States, approximately 6.7 million individuals over the age of 20 have HF, and this number is projected to rise to 8.5 million by 2030. The lifetime risk of developing HF now approaches 24%, or approximately 1 in 4 individuals [3].

Despite advances in medical therapy, end-stage heart failure continues to have a poor prognosis, and heart transplantation remains the gold standard treatment for eligible patients. However, due to the scarcity of donor hearts and the growing number of patients with advanced HF, alternative strategies are necessary to bridge patients to transplantation or provide durable support [4]. Mechanical circulatory support (MCS) devices, particularly left ventricular assist devices (LVADs), have revolutionized the management of patients with end-stage HF, offering improved survival and quality of life [5]. Originally introduced as a bridge to transplantation, left ventricular assist devices have also become an established destination therapy option for patients with end-stage heart failure who are not candidates for heart transplantation [6].

The evolution of LVAD technology, including the development of continuous-flow and fully magnetically levitated devices like the HeartMate 3 (Abott, Chicago, IL, USA) has significantly reduced device-related complications such as pump thrombosis and stroke [7]. Despite these advancements, significant challenges persist, including infection, bleeding, arrhythmias, and particularly right ventricular failure. Right ventricular failure following LVAD implantation is associated with increased morbidity, prolonged hospitalization, and may contribute to adverse post-transplant evolution through pretransplant systemic dysfunction. [5,7,8].

Patient selection for LVAD therapy remains critical to minimize complications and optimize outcomes. Identifying patients at risk of developing RVF and implementing perioperative management strategies are essential components of care. Moreover, the timing of transplantation after LVAD implantation must balance the benefits of hemodynamic stabilization with the risks associated with prolonged device support [9].

In this report, we present a challenging case of a patient with post-myocarditis dilated cardiomyopathy who developed severe right ventricular failure following LVAD implantation but was successfully bridged to heart transplantation.

## Case presentation

A 46-year-old male, with a history of post-myocarditis dilated cardiomyopathy diagnosed 10 years earlier, presented with advanced heart failure (NYHA class IV) despite guideline-directed medical therapy. His medical history was notable for moderate-to-severe mitral regurgitation, severe tricuspid regurgitation, postcapillary medium-severe pulmonary hypertension combined with pre - and postcapillary increased vascular pulmonary resistances, type 2 diabetes mellitus, and non-sustained ventricular tachycardia managed with a Medtronic Protecta single-chamber implantable cardioverter-defibrillator (ICD) (Medtronic, Minneapolis, USA) implanted in 2018 for primary prevention of sudden cardiac death.

**Fig. 1.**
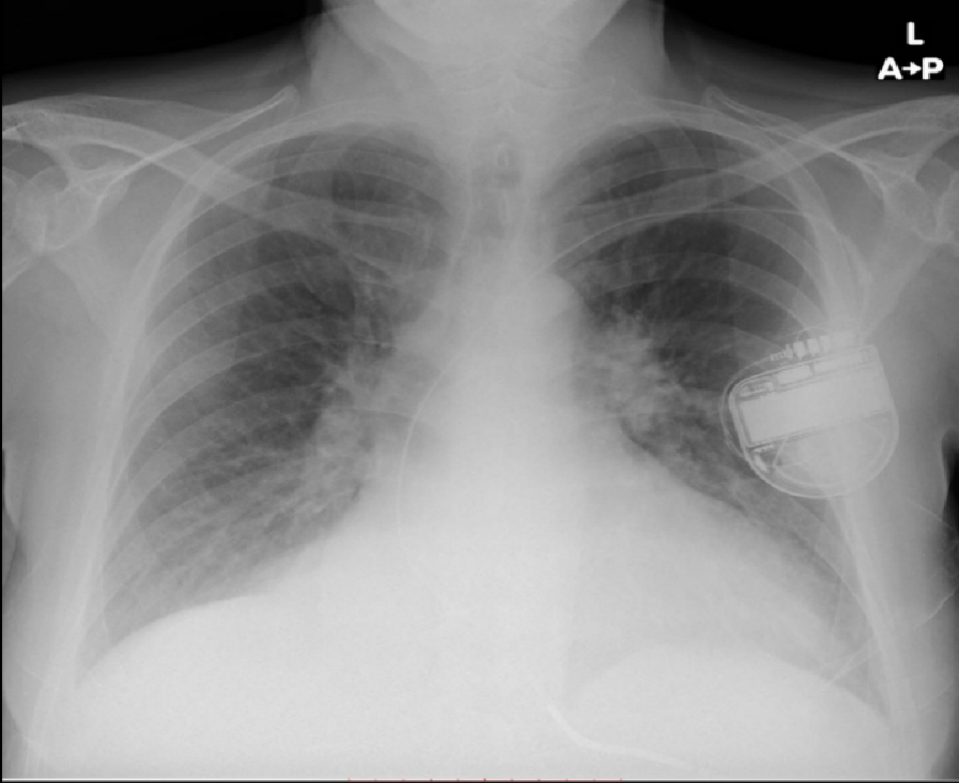
Chest radiography on admission: Marked cardiomegaly with increased interstitial markings, permanent ICD device in situ, without evidence of pulmonary consolidation or pleural effusion

The patient experienced multiple previous hospitalizations for acute decompensated heart failure, with the most recent in January 2023. Upon admission to our institution, he exhibited severe exertional dyspnea, hypotension (BP 92/71 mmHg), tachycardia (HR 118 bpm), and signs of low cardiac output. Laboratory studies revealed elevated lactate and bilirubin levels, indicating metabolic acidosis and early multiorgan dysfunction. Chest radiography showed cardiomegaly without pulmonary consolidation (Figure 1).

**Fig. 2.**
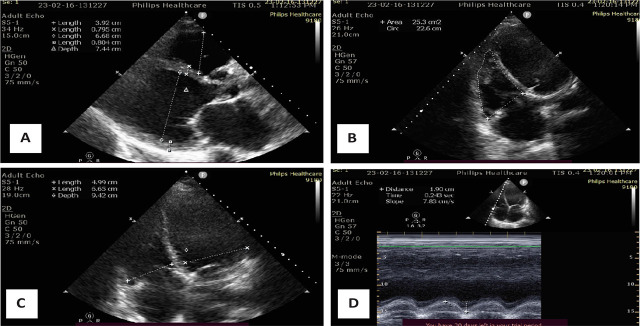
Transthoracic echocardiography on admission: A - Severely dilated left ventricle (LVEDD 66 mm), severely reduced ejection fraction (EF 21%); B - RV FAC – Right ventricle fractional area shortening (28%); C - RV/LV - mid linear dimension ratio in 4 chamber view (0,75); D - TAPSE – tricuspid annular plane systolic excursion (16 mm).

On admission, prior to LVAD implantation, transthoracic echocardiography revealed a severely dilated left ventricle (LVEDd: left ventricular end-diastolic diameter, 66 mm) with markedly reduced systolic function (LVEF: left ventricular ejection fraction by Simpson biplane, 21 %). The right ventricle was also dilated (RVED1: right ventricular end-diastolic diameter, 50 mm) with reduced systolic performance, as indicated by a tricuspid annular plane systolic excursion (TAPSE) of 16 mm and a right ventricular fractional area change (RV FAC) of 28%. Both atria were enlarged: right atrial (RA) area measured 26.5 cm^2^, left atrial (LA) anterior-posterior diameter was 54 mm, and the left atrial volume index (LAVI) was elevated at 59 mL/m^2^. Significant valvular pathology included severe tricuspid regurgitation (peak gradient: 41 mmHg) and moderate-to-severe mitral regurgitation. The estimated pulmonary artery systolic pressure was elevated, suggestive of secondary pulmonary hypertension. (Figure 2).

Right heart catheterization revealed severe pulmonary hypertension, with a mean pulmonary artery pressure (PAPm) of 56 mmHg and a pulmonary capillary wedge pressure (PCWP) of 27 mmHg. The pulmonary vascular resistance (PVR) was 5 Wood units, with a pulmonary vascular resistance/systemic vascular resistance (PVR/SVR) ratio of 0.17, and a transpulmonary pressure gradient (TPG) of 9 mmHg, suggesting combined pre- and post-capillary pulmonary hypertension. These findings, together with the clinical deterioration, define a high-risk hemodynamic profile characterized by progressive biventricular dysfunction and an increased likelihood of right ventricular failure in the context of LVAD therapy.

Despite escalating pharmacologic support—including continuous intravenous administration of dobutamine and dopamine, along with a 24-hour infusion of levosimendan—the patient’s clinical and metabolic condition progressively deteriorated. By hospital day 7, he developed refractory low cardiac output syndrome accompanied by worsening respiratory distress, ultimately requiring endotracheal intubation and mechanical ventilation. At this point, the patient fulfilled the criteria for INTERMACS Profile II, characterized by a declining trajectory in patients dependent on inotropic support, with signs of end-organ dysfunction and imminent risk of hemodynamic collapse. This clinical profile, indicative of critical circulatory compromise despite maximal medical therapy, prompted the decision for urgent LVAD implantation.

Given the progressive deterioration, decision was made to implant a HeartMate 3 LVAD as a bridge to transplantation. The procedure was performed under challenging hemodynamic conditions (on triple inotropic support and vasopressors). The surgical technique follows a standardized approach through median sternotomy, under cardiopulmonary bypass with central arterial and venous cannulation. After systemic heparinization and institution of bypass, the left ventricular apex is identified and selected as the site for inflow cannula insertion. Apical coring is performed, and a sewing ring is secured to the myocardium using pledgeted horizontal mattress sutures. The LVAD inflow cannula is inserted and connected to the ring, ensuring proper alignment.

**Fig. 3.**
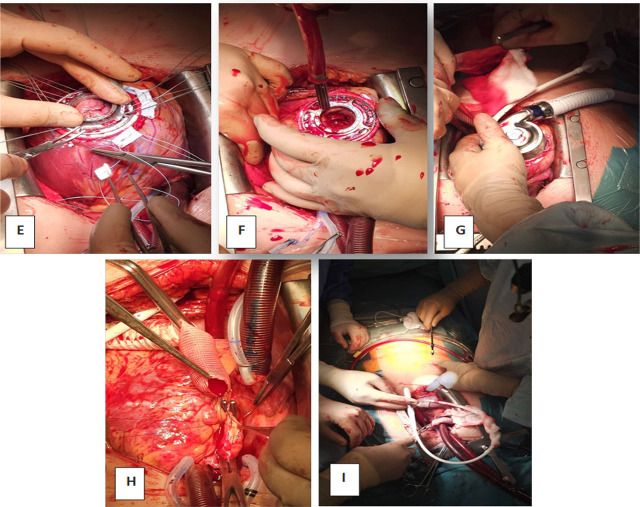
Intraoperative view during LVAD implantation: E - The sewing ring is secured to the myocardium using multiple pledgeted horizontal mattress sutures placed circumferentially; F - A coring device is used to create an opening at the apex; G - The LVAD inflow cannula is inserted through the apical opening and secured to the sewing ring; H - The outflow graft is measured and trimmed to the appropriate length, then anastomosed end-to-side to the ascending aorta using a partial occlusion clamp; I - The driveline is passed through the subcutaneous tissue and brought out through the abdominal wall.

The outflow graft is measured, trimmed, and anastomosed end-to-side to the ascending aorta using a partial clamp. The system is de-aired, and the pump is gradually started. Weaning from cardiopulmonary bypass is performed under echocardiographic and hemodynamic guidance. (see Figure 3).

Postoperatively, the patient developed persistent severe right ventricular failure, requiring high-dose inotropes: Milrinone (Primacor, Sanofi, UK); Noradrenaline (Hameln, GmbH, Germany); Adrenaline(Hameln, GmbH, Germany) and inhaled nitric oxide for right ventricular support. Despite progressive reduction of vasopressors over time, complete weaning of inotropes was not achieved due to irreversible right ventricular dysfunction. Echocardiography post-LVAD showed a dilated LV with visible inflow cannula, severely impaired RV function with a TAPSE of 10 mm, moderate mitral and tricuspid regurgitation, and leftward septal shift. Pump parameters were stable at 4500 rpm, with a flow of 3.5 L/min.

**Fig. 4.**
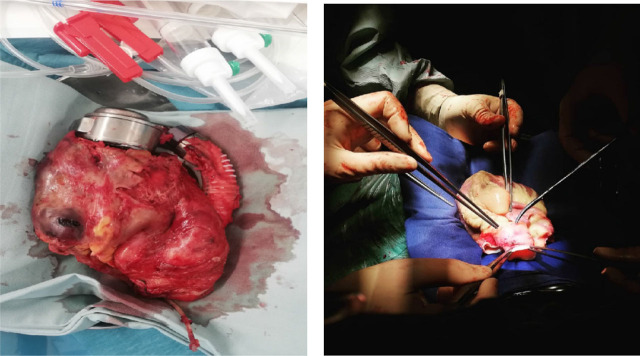
Left picture - Explanted HeartMate 3 LVAD and native heart; Right picture - preparation of the donor heart for implantation.

Given the severe and persistent right ventricular dysfunction, the patient was listed urgently for heart transplantation. After LVAD implantation, he underwent orthotopic heart transplantation. Donor evaluation revealed a RADIAL score indicating a moderate risk of primary graft dysfunction (30%). Extensive adhesiolysis was required during the transplant surgery due to prior LVAD implantation (Figure 4).

Post-transplant, the patient had a favorable evolution: he was extubated on postoperative day 3, inotropic support was discontinued by day 4, and he was discharged from the intensive care unit on day 13. Final hospital discharge occurred 44 days after the transplant.

Follow-up evaluations showed good graft function (EF 50–55%) without significant valvular regurgitation. At 4 months post-transplant, he developed immunosuppression-related complications, including severe neutropenia and acute kidney injury, which were successfully managed with adjustment of immunosuppressive therapy. At 6 months, he was asymptomatic, with normal cardiac function (ISHLT grade 0 biopsy). At 7 months, he experienced an episode of lobar pneumonia, successfully treated with antibiotics. At 2 years and 4 months post-transplant, the patient is in excellent clinical condition, with no complications requiring hospitalization or specific treatment. The evolution has been favorable. The patient has been reintegrated into society and maintains an active lifestyle. Table 1 summarizes the timeline of major clinical events. Table 1 summarizes the timeline of major clinical events.

**Table 1.** Timeline of Clinical Events

**Day / Time Point**	**Event / Intervention**
05.01.2023	Re-hospitalization for acute heart failure, initiation of inotropic support
15.02.2023	Transfer to our center on Dobutamine and Furosemide continuous infusion
Admission Day	Severe hemodynamic instability, metabolic acidosis, renal dysfunction
Day 2–3	Progressive decline despite escalation of inotropes; start of Levosimendan
Day 5	Development pleural effusion; right thoracentesis (520 mL evacuated)
Day 7	Worsening hemodynamics, need for non-invasive ventilation (CPAP)
Day 8	Intubation, mechanical ventilation, renal replacement therapy initiated
Day 10	INTERMACS II profile confirmed
Day 11	HeartMate 3 LVAD implantation
Post-op Day 1–8	Persistent right ventricular failure, high-dose inotropic and vasopressor support
Post-op Day 22–25	Severe RV dysfunction persists; recurrent arrhythmias; hemodynamic instability
Day 27	Heart transplantation performed
Post-TX Day 3	Successful extubation
Post-TX Day 4	Weaning off inotropes
Post-TX Day 13	ICU discharge
Post-TX Day 44	Hospital discharge
4 Months Post-TX	Severe neutropenia, acute kidney injury; management and recovery
6 Months Post-TX	Asymptomatic, stable graft function (ISHLT 0)
7 Months Post-TX	Lobar pneumonia; antibiotic treatment and recovery
2 Years post-TX	Excellent clinical condition, active lifestyle

CPAP- continuous positive airway pressure; LVAD- left ventricle assist device; RV- right ventricle; ISHLT- International Society for Heart and Lung Transplantation; TX- heart transplant.

## Discussions

Heart failure represents a major global health burden, with over 64 million patients affected worldwide. Despite therapeutic advances, HF remains associated with significant morbidity and mortality [1,2,3]. For patients with end-stage heart failure refractory to medical therapy, heart transplantation is the gold standard. However, the shortage of donor organs necessitated the development of mechanical circulatory support devices, particularly left ventricular assist devices, as a bridge to transplantation (BTT) strategy [4]. In our case, the patient with dilated cardiomyopathy secondary to post-myocarditis heart failure, complicated by severe mitral and tricuspid regurgitation, was implanted with a HeartMate 3 LVAD due to progressive hemodynamic deterioration and multiorgan dysfunction, reaching an INTERMACS II profile.

Continuous-flow LVADs, especially the HeartMate 3, have significantly improved survival and quality of life compared to earlier devices [5]. The MOMENTUM 3 trial demonstrated superior outcomes with HeartMate 3, showing a 79% survival at 2 years without disabling strokes or pump replacement compared to HeartMate II [6,7]. Nevertheless, LVAD therapy is not without complications, including bleeding, infections and device malfunction [8,9]. Our patient developed persistent severe RV dysfunction after LVAD implantation, consistent with findings from other cohorts reporting RVF in up to 40% of patients [10].

Its pathophysiology involves increased preload to the RV, altered septal geometry due to LV unloading, and elevated pulmonary vascular resistance. Studies by Kormos et al. have identified key preoperative risk factors, including elevated central venous pressure, impaired RV function, and severe tricuspid regurgitation [11,12,13,14]. In our case, pre-existing tricuspid regurgitation and pulmonary hypertension likely contributed to RVF development. In our patient, pre-existing tricuspid regurgitation and pulmonary hypertension likely predisposed to RV failure. Persistent RVF has been associated with worse outcomes, as demonstrated by Siems et al., who highlighted the strong correlation between RVF and prolonged hospital stays and decreased survival [8,15]. Strategies such as early identification of high-risk patients, aggressive pre-LVAD optimization, and prompt right ventricular mechanical support when needed, as emphasized by Lo Coco et al., are crucial. The use of pulmonary vasodilators and inotropic agents can improve right ventricular performance pre-and post-implantation [13,16,17]. Despite the presence of preoperative risk factors for right ventricular failure following LVAD implantation, the patient was weaned from cardiopulmonary bypass without high-dose inotropic or vasoactive support and demonstrated stable hemodynamics upon pump removal. These objective findings argued against immediate placement of a right ventricular assist device. When right ventricular failure subsequently developed, we considered two primary management strategies: implantation of a dedicated right ventricular mechanical support device or proceeding to orthotopic heart transplantation, which would address both left and right ventricular dysfunction. Although donor organs are in limited supply, our patient fortunately received a transplant after a very brief waiting period.

Timing of heart transplantation after LVAD implantation significantly impacts outcomes. Brown et al. found that transplantation within 30 days of LVAD placement was associated with higher mortality, whereas delaying transplantation improved survival. Similarly, Garbade et al. recommended allowing for stabilization post-LVAD before proceeding to transplant. However, even in patients with LVAD support of less than 30 days who develop severe right ventricular failure, heart transplantation an optimal therapeutic strategy [10,16,18,19]. In our case, transplantation was performed approximately one month after LVAD implantation, aligning with these recommendations and likely contributing to a favorable postoperative course.

Beyond hemodynamic considerations, psychosocial assessment is critical for patient selection and outcomes. Dew et al. highlighted the need for comprehensive evaluation of psychosocial factors such as treatment adherence, mental health, and social support, which are associated with post-implantation and post-transplant survival [20]. Addressing these factors early can improve both short- and long-term outcomes.

Technological improvements, such as the HeartMate 3’s artificial pulsatility and hemocompatibility, have helped reduce complications like pump thrombosis and stroke. Yet, challenges like driveline infections and gastrointestinal bleeding persist [21]. Future directions include the development of fully implantable systems and better predictive models for RV failure [22].

## Conclusion

Our case underscores several crucial points identified across the literature: the necessity for careful preoperative risk assessment, the importance of stabilization before transplantation, and the impact of RVF on outcomes. Heart transplantation, performed after adequate stabilization on LVAD support, remains the definitive treatment, offering the best long-term outcomes for patients with advanced heart failure.
